# The relationship between parental phubbing and preschool children’s social–emotional competence: a serial mediation model of family intimacy and parent–child attachment

**DOI:** 10.3389/fpsyg.2026.1850205

**Published:** 2026-08-21

**Authors:** Jun Liu, Jiani Rong, Longfeng Zhu, Yixin Zhang

**Affiliations:** 1 School of Teacher Education and Development, Fujian Preschool Education College, Fuzhou, China; 2 Institute of Curriculum and Instruction, East China Normal University, Shanghai, China; 3 School of Preschool Education, Fuyang Preschool Teachers College, Fuyang, China; 4 Faculty of Educational Sciences, University of Helsinki, Helsinki, Finland

**Keywords:** family intimacy, parental phubbing, parent–child attachment, preschool children, social–emotional competence

## Abstract

Parental phubbing, a growing phenomenon in the digital era, has raised concerns about its impact on young children’s development. However, little is known about how parental phubbing influences preschool children’s social–emotional competence and the underlying family mechanisms. Drawing on social learning theory, attachment theory, and family systems theory, this study examines the mediating roles of family intimacy and parent–child attachment. A total of 1,113 parents of preschool children in China participated in this study. The results showed that parental phubbing was negatively associated with children’s social–emotional competence. First, family intimacy mediated the relationship between parental phubbing and children’s social–emotional competence. Second, parent–child attachment also served as a mediator. More importantly, a significant serial mediation pathway was identified, whereby parental phubbing reduced family intimacy, which in turn weakened parent–child attachment and ultimately impaired children’s social–emotional competence. These findings highlight the importance of family processes in understanding the negative effects of parental phubbing and provide practical implications for promoting young children’s social–emotional development.

## 1 Introduction

According to the CASEL framework, social–emotional competence involves individuals’ ability to recognize and regulate their own emotions, perceive and respond to others’ emotions, set goals, solve problems, and master social rules and interpersonal skills which are needed for maintaining healthy relationships (Denham et al., 2003; Pentón Herrera, 2020). For young children, social–emotional competence is essential for adapting to their environments, transitioning smoothly into primary schooling, and achieving coordinated physical and mental development (Denham et al., 2003; Elias et al., 1997; Jones et al., 2015). It is also the key predictor to success in school and future life (Jones et al., 2015). Early childhood is a critical period for the development of social–emotional competence, and identifying factors influencing children’s social–emotional capacities is particularly important in the era of artificial intelligence.

Existing research has extensively examined the factors influencing the development of young children’s social–emotional competence. The family and the school environments exert direct and foundational influences. Parent–child relationships, parenting styles (Bronfenbrenner and Morris, 2007; Deleş and Aral, 2026), teacher-child relationships and peer interactions represent key determinants of children’s social–emotional growth (Ladd, 2005; Sabol and Pianta, 2012). Although prior studies have examined various predictors of social–emotional competence, such as family socioeconomic status, peer acceptance, and exposure to family adversity (Evans et al., 2013), relatively little attention has been paid to the mechanisms through which parental phubbing influences young children’s social–emotional competence.

With the deep integration of the internet into family life, parental phubbing has become increasingly prevalent. This behavior not only reduces parent–child interaction time but may also alter young children’s perception of the quality of family relationships. Within the Chinese cultural context, child development is typically understood through the lens of family relationships and educational responsibilities. Parents are not only expected to fulfill basic caregiving duties but also are entrusted with providing multifaceted support encompassing emotional companionship, behavioral guidance, and habit formation. The Family Education Promotion Law of the People’s Republic of China advocates for fostering a positive and harmonious family environment, which serves as the crucial foundation for achieving high-quality family education and promoting children’s holistic development. Examining the impact of parental phubbing on young children’s social–emotional competence within the context of Chinese family education helps to reveal the potential risks which changes in family interaction in the digital era pose to child development.

Drawing on the holistic perspective of Family Systems Theory, this study conceptualizes the family as a dynamically balanced system. From this perspective, parental phubbing can be regarded as a behavior which disrupts internal interactions and emotional connections within the system (Cox and Paley, 1997; Minuchin, 1974). On this basis, the study aims to investigate how this behavior, by affecting the emotional climate at the family system level, namely family intimacy, the dyadic parent–child relationship within the core subsystem which is parent–child attachment, and influences the development of young children’s social–emotional competence. Accordingly, family intimacy and parent–child attachment are incorporated into the model as mediating variables, with the goal of elucidating the underlying mechanisms through which family factors affect young children’s social–emotional development.

### 1.1 The relationship between parental phubbing and social–emotional competence

Parental phubbing refers to situations in which parents excessively focus on electronic devices during parent–child interactions or in the family environment, diverting their attention away from their children and exhibiting behaviors such as distraction, emotional disengagement, or neglect of children’s needs (McDaniel and Radesky, 2018). Research has indicated that parental phubbing can undermine young children’s self-esteem and core self-evaluation (Lin et al., 2025; McDaniel and Coyne, 2016; Mun, 2024), increase problems such as anxiety, depression, anger, and self-isolation (Bai et al., 2020; Coyne et al., 2021), and is detrimental to their interpersonal functioning (Wang X. et al., 2022).

From the perspective of Family Systems Theory, parental phubbing acts as a disruptive “input” within the system, reducing the opportunities for young children to acquire effective social learning from their parents. Social Learning Theory provides an explanation for this direct influence pathway. Children learn through observing and imitating others, especially their parents, and acquire skills such as emotion recognition, emotion regulation, and interpersonal communication through parent–child interactions (Bandura, 1977; Morris et al., 2007). When parents are preoccupied with mobile devices and respond less sensitively, children lose opportunities to learn constructive emotional regulation strategies through parental modeling. Meanwhile, children may unconsciously imitate their parents’ device-use behaviors, potentially increasing their risk of problematic electronic device use and behavioral difficulties (Coyne et al., 2021), which will thereby exert a detrimental impact on the development of children’s social competence. The Alternative Hypothesis suggests that parents’ overreliance on electronic devices reduces the amount of face-to-face interaction with their children. Such direct interaction is foundational for developing and regulating social–emotional abilities, and reductions in high-quality parent–child interaction because of parental phubbing may negatively affect children’s social–emotional competence (Domoff et al., 2019).

Based on these findings, this study proposes Hypothesis 1: Parental phubbing is negatively associated with young children’s social–emotional competence.

### 1.2 The mediating role of family intimacy between parental phubbing and young children’s social–emotional competence

Family intimacy reflects the frequency and emotional closeness of interactions among family members, including perceived emotional distance, shared time, and consistency in communication patterns (Schrodt et al., 2009). Family Systems Theory posits that family functioning is contingent upon the holistic environmental quality of interactions and emotional connections among members. Parental phubbing, conceptualized as an interactive disruption, attenuates emotional bonds within the family, diminishes the emotional support accessible to children, and conduces to lower levels of family intimacy (Kildare and Middlemiss, 2017), all of which thereby might precipitate a spectrum of internalizing psychological difficulties and externalizing behavioral problems in children (Xie et al., 2019). Children who are repeatedly exposed to parental phubbing may come to feel ignored or excluded by their families and may even develop a belief that the phone is more important than themselves. Such perceptions can evoke feelings of loneliness, worthlessness, and emotional disconnection from family members, thereby reducing family intimacy.

Person-Environment Interaction Theory further elucidates the critical role of this environmental variable, individuals’ behaviors and attitude are shaped by the interplay between personal characteristics and environmental contexts (Sameroff, 2010). Under this framework, family intimacy constitutes the “environmental context” in which young children’s social learning takes place. Appropriate levels of family intimacy provide children with a secure emotional base from which they can explore the world, foster social exploration and reduce behavioral problems (Morris et al., 2007). As the key settings for emotional expression and psychological interaction among family members, a highly intimate family environment protects children from the negative impact of adverse conditions and enhances their emotional regulation abilities (Fosco and Grych, 2008), and enhances emotional regulation abilities. Such environments also promote children’s engagement in social interactions beyond the family and facilitate the generalization of prosocial behaviors to broader contexts. In contrast, children raised in low-intimacy family environments tend to receive less parental warmth and support, experience more negative emotions, and exhibit poorer emotional regulation, which may hinder their academic engagement and social functioning (Eisenberg et al., 2001; Repetti et al., 2002). Additionally, a warm and cohesive family climate has been consistently linked to adolescents’ psychological well-being and social adjustment (Olson, 2000; Steinberg and Morris, 2001).

Based on these findings, this study proposes Hypothesis 2: Family intimacy mediates the relationship between parental phubbing and young children’s social–emotional competence.

### 1.3 The mediating role of parent–child attachment between parental phubbing and young children’s social–emotional competence

Parent–child attachment refers to a stable and enduring emotional bond between children and their caregivers, typically characterized by children’s desire for the maintenance of closeness to attachment figures (Ainsworth et al., 1978). Existing research shows that attachment formation is influenced by both parenting practices and family system characteristics (Belsky, 1984; De Wolff and Van Ijzendoorn, 1997). This provides a key rationale for incorporating parental phubbing (representing parenting practices) and family intimacy (representing family system climate) into the analytical framework of the present study. Attachment relationships further influence children’s self-perceptions, environmental interpretations, and interpersonal orientations (Sroufe, 2005).

Existing studies have shown that parental phubbing is negatively associated with parent–child attachment. Within the family system, the parent–child relationship is a core subsystem. When parents are distracted by mobile devices, their responsiveness and interaction frequency decrease, undermining the quality of parent–child interactions and hindering the development of secure attachment. Moreover, when children attempt to gain attention during parental device use, parents may respond with irritation or negativity, which can exacerbate parent–child conflict and further disrupt attachment formation (Stockdale et al., 2018).

Attachment theory emphasizes that the formation of secure parent–child attachment depends on parents’ sensitive perception and timely response to children’s signals, and specifically elucidates how the quality of this subsystem connection affects individual development (Biringen, 2000). Secure attachment has been associated with fewer emotional and behavioral problems and better social functioning in children, stronger interpersonal relationships, and better social–emotional development (Fearon et al., 2010). When the overall emotional bonding within the family is weakened by parental phubbing, the consistency of parents’ responses to children’s emotions and behaviors during parent–child interactions is compromised, hindering the establishment of secure attachment in young children. In contrast, insecure attachment is linked to poorer emotional regulation and a higher likelihood of externalizing problems such as aggression and hyperactivity (Bernard et al., 2012; Fearon et al., 2010).

Based on these findings, this study proposes Hypothesis 3: Parent–child attachment mediates the relationship between parental phubbing and young children’s social–emotional competence.

### 1.4 The serial mediating roles of family intimacy and parent–child attachment between parental phubbing and young children’s social–emotional competence

Family systems theory posits that the family operates as an interconnected and dynamically balanced system. Under this framework, children’s development is not isolated from individual caregiving behaviors but is rooted in the dynamic balance of the entire family system, which supports emotional functioning and social adaptation. Children benefit most only when high-quality emotional synergy and supportive interactions are maintained among subsystems within the family (Cox and Paley, 1997; Minuchin, 1974).

Parental phubbing, as a negative form of technology-related disruption in family interactions, serves as an initial input which disrupts the system’s balance, undermining the overall emotional climate and supportive environment of the system (McDaniel and Coyne, 2016; Stockdale et al., 2018). Furthermore, the deterioration of the overall system environment further reduces family intimacy. Family intimacy, in turn, provides a critical emotional foundation for the development of parent–child attachment. When parental engagement is reduced due to device use, children’s willingness to communicate may decline, weakening their sense of family belonging and increasing the likelihood of insecure attachment (Przybylski and Weinstein, 2013).

Parent–child attachment serves as a protective factor for children’s emotional security and is fundamental to early social–emotional development, with its quality directly influencing children’s emotion regulation abilities, attentional regulation, emotional responsiveness, and adaptive reactions (Dykas and Cassidy, 2011; Fearon et al., 2010; Thompson, 2008). In summary, parental phubbing may undermine young children’s social–emotional competence by disrupting family system balance, thereby reducing family intimacy and subsequently impairing the quality of parent–child attachment. Accordingly, this study proposes Hypothesis 4: Family intimacy and parent–child attachment jointly exert a serial mediating effect between parental phubbing and young children’s social–emotional competence.

The core theoretical proposition of this study holds that family functions as an integrated system, and its impacts on preschool children are realized via the hierarchical pathway: overall environmental quality represented by family intimacy → core subsystem function reflected by parent–child attachment → individual development manifested as social–emotional competence. As a negative external input within the family system, parental phubbing can disrupt this entire developmental process in a top-down manner.

### 1.5 Hypotheses

**Figure 1:**
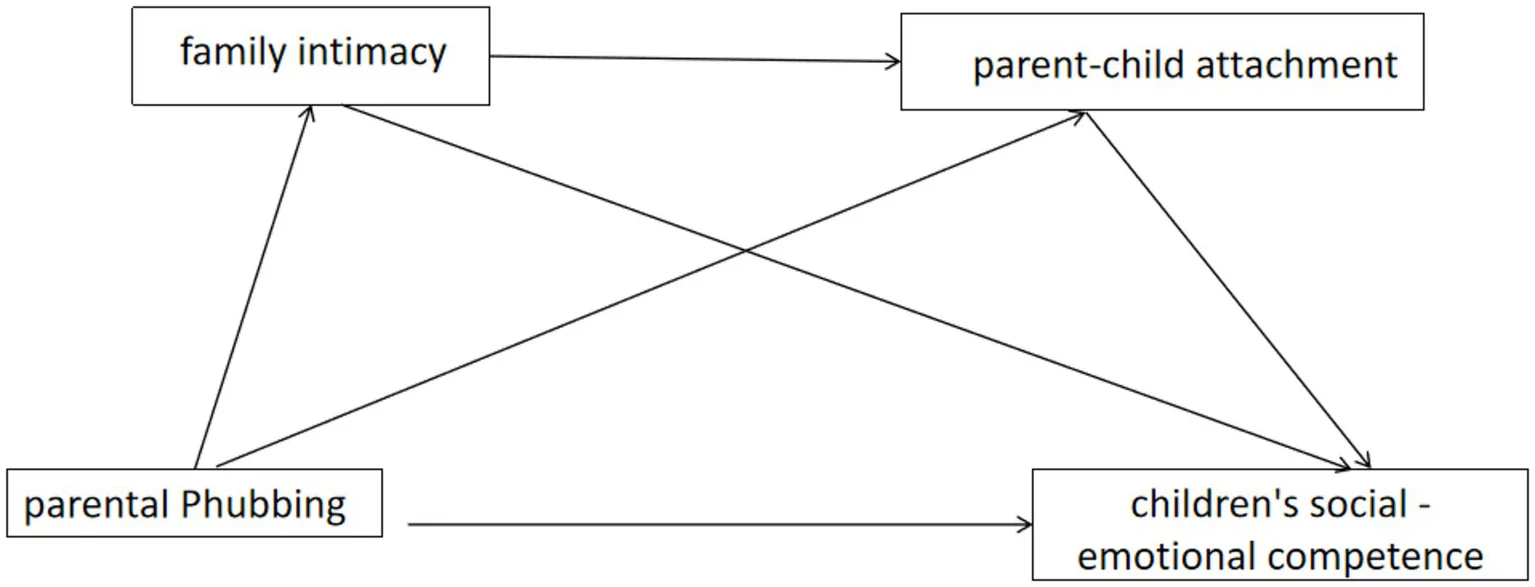
The proposed mediation model.

In this study, a serial mediation model (see Figure 1) was constructed to examine the mechanisms through which parental phubbing influences young children’s social–emotional competence via family intimacy and parent–child attachment. Based on relevant theoretical and empirical evidence, the following hypotheses were proposed:

*H1*: Parental phubbing is negatively associated with young children’s social–emotional competence.

*H2*: Family intimacy mediates the association between parental phubbing and young children’s social–emotional competence.

*H3*: Parent–child attachment mediates the association between parental phubbing and young children’s social–emotional competence.

*H4*: Family intimacy and parent–child attachment jointly serve as serial mediators in the association between parental phubbing and young children’s social–emotional competence.

## 2 Materials and methods

### 2.1 Participants and procedure

A cluster sampling method was employed to recruit participants from kindergartens in Fujian Province, China. The selection of these institutions was grounded in three primary considerations: (1) the research team’s accessibility to the site and the resulting practical feasibility; (2) Fujian’s status as a relatively economically developed southeastern coastal province with high digital device penetration, providing an appropriate sociocultural context for examining parental phubbing; and (3) the selected kindergartens’ representativeness in emphasizing social–emotional education, which closely aligns with this study’s focus on children’s social–emotional competence. Following approval and cooperation from kindergarten principals, informed consent forms and questionnaires were distributed by class teachers to all parents of children enrolled in the participating classes. A total of 1,120 questionnaires were distributed, and invalid questionnaires with response times that were too short or too long or showed patterned responses were excluded, and 1,113 valid questionnaires were returned, yielding an effective response rate of 99.37%. Among the participating children, 576 were boys (51.8%) and 537 were girls (48.2%). Regarding the participating parents, 245 were fathers (22%) and 868 were mothers (78%).

The study was approved by the Biomedical Research Ethics Committee of Fujian Medical University (Approval No. 2025-283), The inclusion criteria are parents of preschool children aged 3–6 who are enrolled in kindergartens in Fujian Province. Prior to data collection, written informed consent forms were distributed to all parents of young children, clearly stating the research purpose, procedures, data usage, and confidentiality principles. Participation was explicitly voluntary, and participants could withdraw unconditionally without affecting their children’s rights or benefits. The questionnaires were designed to be anonymous, with no collection of personally identifiable information such as names; only codes were used to label kindergartens and classes. The research strictly adhered to the principle of “no harm,” and all items were ethically reviewed to avoid questions which might induce parental anxiety or involve children’s privacy.

### 2.2 Instruments

#### 2.2.1 Parental phubbing

Parental phubbing was measured by using a Chinese revised version of the Partner Phubbing Scale originally developed by Roberts and David (2016) and Zu et al. (2022). The scale consists of 9 items rated on a 5-point Likert scale, with higher scores indicating more frequent parental phubbing behavior. The Chinese version has been previously used in empirical studies and demonstrates acceptable reliability. In the present study, Cronbach’s *α* was 0.791. The model fit indices for scale validity were satisfactory: χ^2^/df = 5.911, CFI = 0.962, TLI = 0.949, RMSEA = 0.066, SRMR = 0.033.

#### 2.2.2 Family intimacy

Family intimacy was assessed by using a Chinese adaptation (Phillips et al., 1999) of the Family Adaptability and Cohesion Evaluation Scales (FACES). The scale includes 16 items rated on a 5-point Likert scale, with higher scores indicating greater family intimacy. The Cronbach’s *α* in this study was 0.881. The model fit indices for scale validity were satisfactory: χ^2^/df = 6.160, CFI = 0.927, TLI = 0.912, RMSEA = 0.068, SRMR = 0.048.

#### 2.2.3 Parent–child attachment

Parent–child attachment was measured by using a Chinese revised version (Olson, 1986; Wang Y. et al., 2022) derived from the Attachment Q-Sort (AQS) developed by Waters and Deane (Waters and Deane, 1985). The scale consists of 18 items rated on a 5-point Likert scale, with higher scores indicating stronger parent–child attachment. The internal consistency in this study was acceptable (Cronbach’s α = 0.800). The model fit indices were acceptable: χ^2^/df = 4.907, CFI = 0.913, TLI = 0.896, RMSEA = 0.059, SRMR = 0.059, with the TLI approaching the conventional 0.90 threshold.

#### 2.2.4 Young children’s social–emotional competence

Children’s social–emotional competence was assessed by using a scale developed for Chinese preschool populations (Cao, 2023), comprising 16 items across three dimensions: self-awareness, interpersonal skills, and self-regulation. These dimensions are consistent with established theoretical frameworks of social–emotional development, particularly the social–emotional learning (SEL) model proposed by CASEL (Pentón Herrera, 2020). All items were rated on a 5-point Likert scale, with higher scores indicating higher levels of social–emotional competence. The scale demonstrated high internal consistency (Cronbach’s α = 0.903). The model fit indices for scale validity were satisfactory: χ^2^/df = 6.850, CFI = 0.922, TLI = 0.904, RMSEA = 0.073, SRMR = 0.048.

### 2.3 Statistical analysis

Data were analyzed using IBM SPSS Statistics 23.0. Descriptive statistics and Pearson correlation analyses were conducted to examine the associations among the study variables.

To test the hypothesized mediation effects, the PROCESS macro (Model 6) developed by Hayes (2013) was employed. Indirect effects were estimated using a bootstrapping procedure with 5,000 resamples, and 95% bias-corrected confidence intervals (CIs) were used to determine statistical significance. Mediation effects were considered significant if the confidence intervals did not include zero (Hayes, 2013).

To assess potential common method bias, Harman’s single-factor test was conducted. The results of an exploratory factor analysis indicated that 11 factors with eigenvalues greater than 1 were extracted, and the first factor accounted for 18.70% of the total variance, which is below the recommended threshold of 40% (Podsakoff et al., 2003). These findings suggest that common method bias was not a serious concern in the present study.

## 3 Results

### 3.1 Descriptive statistics and correlation analysis

The means, standard deviations, and correlations of all variables are presented in Table 1. Correlation analyses showed that parental phubbing was significantly negatively correlated with children’s social–emotional competence, family intimacy, and parent–child attachment. Family intimacy and parent–child attachment were significantly positively correlated with children’s social–emotional competence. In addition, family intimacy was positively correlated with parent–child attachment (*p* < 0.01). The detailed results are shown in Table 1.

**Table 1:** Mean, standard deviation, and correlation matrix of each variable.

Variables	*M* ± *SD*	1	2	3	4
1. Family intimacy	3.931 ± 0.601	1			
2. Children’s social–emotional competence	3.505 ± 0.587	0.398^**^	1		
3. Parental phubbing	2.777 ± 0.583	−0.192^**^	−0.132^**^	1	
4. Parent–child attachment	3.882 ± 0.451	0.325^**^	0.288^**^	−0.232^**^	1

*p < 0.05, **p < 0.01, ***p < 0.001.

### 3.2 Chain mediating effect test

The correlation results met the statistical requirements for conducting mediation analysis. Using the PROCESS macro (Model 6) in SPSS, we tested the mediating roles of family intimacy and parent–child attachment in the relationship between parental phubbing and young children’s social–emotional competence. Following Hayes’ procedures for mediation analysis, parental age, education level, employment status, and child gender were included as control variables.

**Figure 2:**
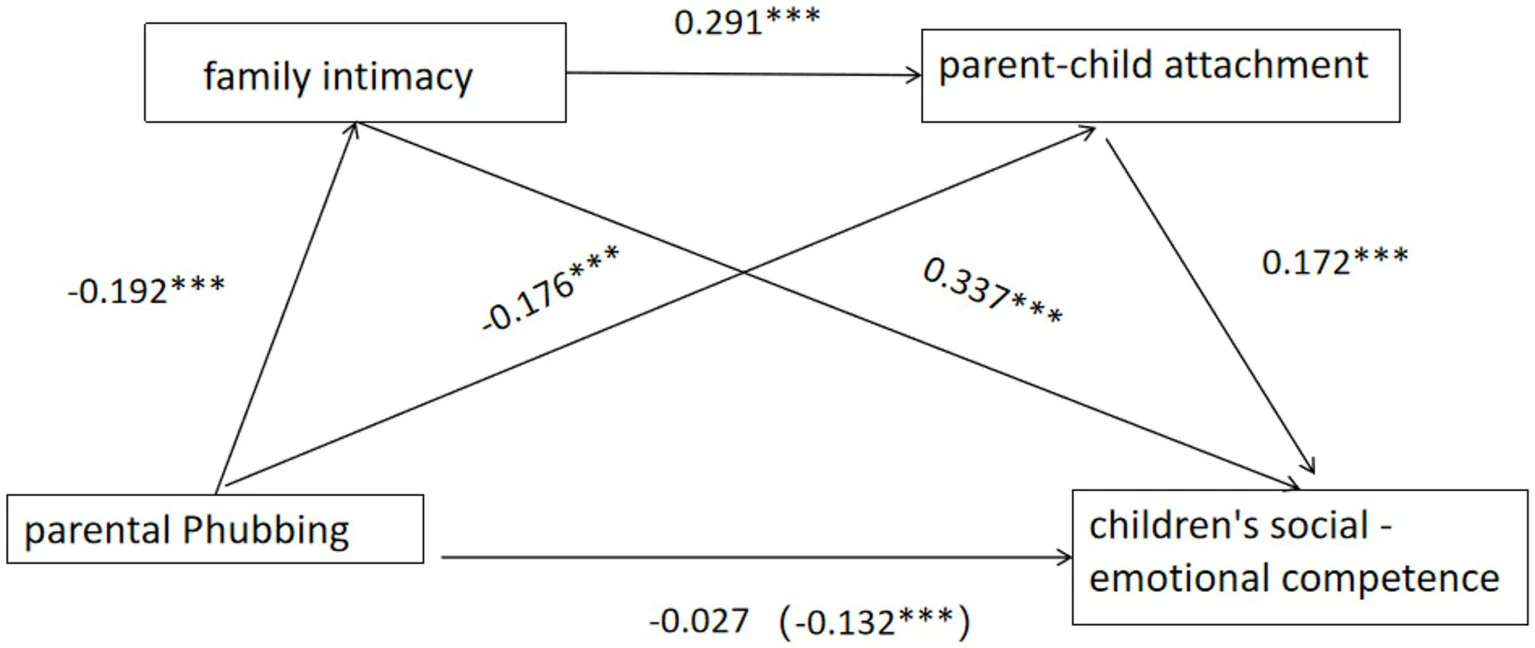
Path model of the effect of parental phubbing on children’s social–emotional competence.*p < 0.05, **p < 0.01, ***p < 0.001.

The results (see Table 2 and Figure 2) showed that parental phubbing significantly negatively predicted children’s social–emotional competence (*β* = −0.132, *t* = −4.437, *p* < 0.001), family intimacy (*β* = −0.192, *t* = −6.521, *p* < 0.001), and parent–child attachment (β = −0.176, *t* = −6.207, *p* < 0.001). When parental phubbing, family intimacy, and parent–child attachment were simultaneously entered into the model, the direct effect of parental phubbing on children’s social–emotional competence became nonsignificant (*β* = −0.027, *t* = −0.970, *p* > 0.05). Family intimacy significantly predicted parent–child attachment (*β* = 0.291, *t* = 10.241, *p* < 0.001), and both family intimacy (*β* = 0.337, *t* = 11.679, *p* < 0.001) and parent–child attachment (*β* = 0.172, *t* = 5.919, *p* < 0.001) significantly positively predicted children’s social–emotional competence.

**Table 2:** Regression analysis of the relationships among model variables.

Outcome variable	Predictor	*R*	*R* ^2^	*F*	*β*	*t*
Family intimacy	Parental phubbing	−0.192	0.037	42.525	−0.192	−6.521^***^
Parent–child attachment	Parental phubbing	0.368	0.136	87.116	−0.176	−6.207^***^
	Family intimacy				0.291	10.241^***^
Children’s social–emotional competence	Parental phubbing	0.433	0.187	85.281	−0.027	−0.970
	Family intimacy				0.337	11.679^***^
	Parent–child attachment				0.172	5.919^***^
Children’s social–emotional competence	Parental phubbing	0.132	0.017	19.691	−0.132	−4.437^***^

All variables in the model were standardized prior to analysis. ^***^p < 0.001.

The mediation analysis (see Table 3) revealed that family intimacy and parent–child attachment exerted a complete mediating effect between parental phubbing and children’s social–emotional competence. The total indirect effect was −0.105 (Boot 95% CI: −0.136 to −0.076), accounting for 79.5% of the total effect (−0.132).

**Table 3:** Direct, indirect, and total effects of parental phubbing on children’s social–emotional competence.

Model pathways	Estimated effect (β)	Boot SE	95% CI (Lower)	95% CI (Upper)	Proportion (%)
Total effect	−0.132	0.030	−0.190	−0.074	100
Direct effect	−0.027	0.028	−0.082	0.028	20.5
Total indirect effect	−0.105	0.016	−0.136	−0.076	79.5
PH → FI → SEC	−0.065	0.013	−0.093	−0.040	49.2
PH → PCA → SEC	−0.030	0.008	−0.047	−0.016	22.7
PH → FI → PCA → SEC	−0.010	0.003	−0.016	−0.005	7.6

PH, parental phubbing; FI, family intimacy; PCA, parent–child attachment; SEC, social–emotional competence. *p < 0.05, **p < 0.01, ***p < 0.001.

Specifically, the mediation analysis revealed three significant indirect pathways. First, parental phubbing affected children’s social–emotional competence through an independent mediating effect of family intimacy (Parental phubbing → Family intimacy → Social–emotional competence), with an indirect effect of −0.065, accounting for 49.2% of the total indirect effect. Second, parent–child attachment also served as an independent mediator (Parental phubbing → Parent–child attachment → Social–emotional competence), yielding an indirect effect of −0.030 and explaining 22.7% of the total indirect effect. Finally, a significant serial mediation pathway was identified in which parental phubbing reduced family intimacy, which subsequently diminished parent–child attachment, ultimately lowering children’s social–emotional competence (Parental phubbing → Family intimacy → Parent–child attachment → Social–emotional competence). This indirect effect was −0.010, accounting for 7.6% of the total indirect effect. The 95% Bootstrap confidence intervals for all three pathways did not include zero, indicating that each indirect effect was statistically significant.

## 4 Discussion

### 4.1 The relationship between parental phubbing and children’s social–emotional competence

The results of the study showed that parental phubbing was significantly negatively correlated with preschool children’s social–emotional competence: the higher the level of parental phubbing, the poorer the social–emotional development of young children. These findings partially support the conclusions of previous studies, parental phubbing was positively associated with social withdrawal and aggression among children aged 4–10 years (Wang X. et al., 2022), and a positive association between parental phubbing and behavioral problems was reported among Italian preschoolers (Puligheddu et al., 2025). Additionally, a meta-analysis found that parental phubbing was negatively associated with children’s social–emotional competence and self-concept and positively associated with internalizing and externalizing problems (Zhang et al., 2023), supporting Hypothesis 1.

Building on these studies, the present study examined social–emotional competence as an integrated developmental outcome and investigated the family relational processes underlying this association. Although recent specific empirical studies have primarily focused on social withdrawal, aggression, or broader behavioral problems, the present study further examined the family relational mechanisms of preschool children’s social–emotional competence.

Drawing on Social Learning Theory and the Alternative Hypothesis, this study posits that parental phubbing may exert harmful effects through the following pathways. Firstly, Social Learning Theory suggests that children acquire social behaviors and emotional skills through observation and imitation (Bandura, 1977). Family members, especially parents serve as primary role models in early childhood, exerting a direct influence on the development of children’s social–emotional competencies. When parents are frequently engaged with electronic devices, their modeling function is diminished, limiting children’s opportunities to observe and learn adaptive social behavior, and also leads young children to acquire the behavioral pattern of attentional disengagement from their parents.

Secondly, face-to-face parent–child interaction is a critical condition for children to acquire social skills; however, parental phubbing deprives young children of time for face-to-face social practice and emotional expression, thereby constraining children’s acquisition of social knowledge and emotional regulation abilities (McDaniel and Radesky, 2018). From this perspective, parental phubbing in Chinese families is not merely ordinary use of electronic devices, but can be perceived by children as insufficient parent–child interaction and a decline in the quality of parental companionship.

Thirdly, parents’ active redirection of attention from their children to digital device’ screens may lead children to experience heightened emotional neglect, and this specific form of relational traumatic experience may disrupt children’s emotion regulation abilities and increase the risk of maladaptive behavioral outcomes (Radesky et al., 2014; Repacholi and Meltzoff, 2007). Compared with children who experience consistent parental engagement, those exposed to higher levels of parental distraction tend to exhibit poorer emotional regulation and greater behavioral difficulties, which in turn undermine their social–emotional competence.

Parental phubbing can therefore be conceptualized as a form of disrupted parenting behavior. Such negative behaviors hinder children’s ability to regulate emotions and increase their susceptibility to emotional instability and problem behaviors when facing challenges (Cho and Lee, 2017; Stockdale et al., 2018).

### 4.2 The mediating role of family intimacy between parental phubbing and children’s social–emotional competence

This research found that family intimacy mediates the association between parental phubbing and children’s social–emotional competence, consistent with prior research demonstrating that family intimacy mediates the relationship between adverse parenting behaviors and children’s adjustment (Song and Wang, 2025), although parental phubbing was not examined in their study, their findings similarly indicate that the family relational climate may influence children’s developmental adjustment. The present study extends this family-process perspective to the relationship between parental phubbing and preschool children’s social–emotional competence supporting Hypothesis 2. This finding supports the path in which parental phubbing acts as a negative “system input,” disrupting the overall family environment and consequently affecting young children’s social–emotional competence. The Person-Environment Interaction Theory posits that children’s development is shaped by the dynamic interplay between their personal characteristics and family contextual factors (Sameroff, 2010). The decline in family intimacy, which serves as the foundational context for social learning and individual-environment interactions, directly limits young children’s opportunities to receive emotional support and social modeling within the family. The quality of intrafamilial relationships exerts a foundational influence on children’s development. Within the family system, parental behaviors such as phubbing can disrupt patterns of interaction and emotional exchange, thereby reducing family intimacy and ultimately influencing children’s social–emotional development (Cox and Paley, 1997).

Specifically, when parents excessively engage with electronic devices during interactions with their children, family intimacy is likely to be weakened. Such behavior reduces both the frequency and quality of parent–child interactions and may undermine children’s sense of belonging and emotional security within the family. Previous research has shown that children’s social development is closely associated with family cohesion and the quality of parent–child communication. In families characterized by high levels of intimacy, relationships tend to be more harmonious, emotional bonds are stronger, and supportive communication fosters mutual understanding and prosocial development (Olson, 2000; Koerner and Fitzpatrick, 2002).

In contrast, families with low levels of intimacy often exhibit deficits in emotional expression, verbal communication, and behavioral interaction. Ineffective communication and limited emotional support may increase children’s risk of social anxiety and hinder their engagement in prosocial behaviors. Moreover, the family emotional climate plays a crucial role in shaping children’s emotion regulation capacities, which in turn influence their ability to adapt to external social environments (Eisenberg et al., 2001; Valiente et al., 2006).

The relatively high mediation proportion of 49.2% suggests that, within the Chinese cultural context, the pathway through which parental phubbing undermines young children’s social–emotional competence by disrupting the overall family emotional atmosphere is particularly pronounced. In Chinese families, where intergenerational co-parenting is prevalent, parental phubbing not only reduces the quality of interactions between parents and children, but may also lead other family members (such as grandparents) to feel neglected, thereby diminishing emotional connections among all family members. Thus, family intimacy is a crucial environmental variable for understanding the mechanism through which parental phubbing exerts its harmful effects, as it reflects how impairment in the overall system function is transmitted to children’s development.

Taken together, the emotional closeness and support provided within the family serve as critical foundations for the development of children’s emotion regulation, social adaptation, and problem-solving abilities.

### 4.3 The mediating role of parent–child attachment between parental phubbing and children’s social–emotional competence

This study found that parent–child attachment mediates the association between parental phubbing and children’s social–emotional competence, thereby supporting Hypothesis 3. The results partially corroborate previous findings that mother–child attachment mediated the association between maternal phubbing and young children’s emotional and behavioral problems (Lv et al., 2022). Although that study focused on emotional and behavioral difficulties rather than social–emotional competence, both studies consistently indicate that attachment quality is an important relational process linking parental phubbing with young children’s developmental adjustment. The present study further extends this attachment mechanism to the domain of preschool children’s social–emotional competence, validating the indirect role of parent–child attachment in this association.

Attachment quality is precisely established through emotional responsiveness in daily parent–child interactions. Specifically, this result supports the pathway through which parental phubbing affects children’s emotion regulation and social adaptation by undermining responsive interactions within the parent–child subsystem. This finding is consistent with Attachment Theory which suggests that it is through consistent emotional responsiveness and high-quality interactions that children develop a sense of emotional security, which serves as a foundation for social–emotional competence in early childhood (Thompson, 2008).

In this process, nonverbal communication behaviors, such as eye contact, physical touch, body orientation, vocal tone, and emotionally responsive interactions, play a crucial role in fostering secure attachment. However, when parents are distracted by digital devices, these interactional processes may be disrupted. This form of parental distraction reduces children’s engagement in interactions and diminishes both the frequency and quality of parent–child subsystem communication (Stockdale et al., 2018), thereby undermining the development of secure attachment.

Specifically, higher levels of parental phubbing are associated with reduced parental attention and responsiveness, as well as increased emotional disengagement. Such disengagement may heighten children’s negative emotional experiences and weaken their sense of emotional security. Children who lack emotional security are more likely to experience difficulties in emotion regulation, social competence, and coping with stress, which ultimately hinders the development of social–emotional competence (Brumariu and Kerns, 2010; Ferreira et al., 2024).

Parental phubbing undermines the quality of parent–child relationships, which serves as the core subsystem, thereby directly affecting the formation of young children’s emotional security and the development of their social–emotional competence.

### 4.4 The serial mediating role of family intimacy and parent–child attachment between parental phubbing and children’s social–emotional competence

This study identified a significant positive association between family intimacy and parent–child attachment, which is consistent with attachment theory and prior empirical findings (Ainsworth et al., 1978; Bowlby, 1982). Family intimacy provides a crucial emotional context that facilitates the development of secure parent–child attachment, whereas secure attachment, in turn, reinforces emotional cohesion within the family system.

Two mechanisms may account for this association. First, families characterized by high intimacy are more likely to effectively manage conflicts and challenges in parent–child interactions, thereby maintaining stable attachment relationships. Second, when children perceive warmth, care, and a sense of belonging within the family environment, they experience enhanced psychological security and emotional support. This, in turn, promotes open emotional communication, facilitates harmonious interactions, and contributes to children’s social–emotional competence (Denham, 2006; Morris et al., 2007).

A notable finding of this study is that, after including family intimacy and parent–child attachment as mediators, the direct effect of parental phubbing on children’s social–emotional competence became nonsignificant. Rather, parental phubbing operated indirectly through a sequential mediation process whereby family intimacy shaped parent–child attachment, which subsequently influenced children’s social–emotional competence, thus supporting Hypothesis H4. The sequential pathway identified in the present study echoes recent research that has examined sequential mechanisms linking parental phubbing with preschoolers’ developmental adjustment. Parental phubbing was found to be indirectly associated with preschoolers’ social withdrawal through the sequential pathway of “parent–child conflict → children’s negative emotions” (Zhang and Wang, 2025). Their findings suggest that parental phubbing may be linked to children’s developmental adjustment through interrelated family relational processes. Building on this, the present study extended the sequential mechanism from “parent–child conflict → negative emotions” to a different family relational pathway, namely “family cohesion → parent–child attachment.”

It is evident that the core harmful mechanism of parental phubbing on children’s development does not stem from simple encroachment on interaction time or modeling of undesirable behaviors, but rather from its profound disruption of overall family functioning and relational subsystems.

This finding is consistent with Family Systems Theory, which posits that both the overall functioning of the family system and the dynamic interactions among its subsystems jointly shape children’s development. The overall functioning of the family system takes precedence over its subsystems and influences individual developmental outcomes through hierarchical transmission. Children’s social–emotional competence is not solely determined by direct parental behaviors but is shaped through multi-level processes in which systemic factors such as family intimacy influence core relational subsystems represented by parent–child attachment.

Chinese culture emphasizes that “harmony in the family leads to prosperity in all affairs,” and the overall harmony of the family is regarded as the cornerstone of individual development. High levels of family intimacy may buffer the negative impact of parental phubbing by fostering a warm and supportive emotional climate, thereby enhancing children’s sense of security and promoting adaptive social–emotional development. At the same time, secure parent–child attachment strengthens children’s emotional resilience and trust, enabling them to cope more effectively with external stressors. In turn, this process mitigates the adverse consequences associated with declines in family intimacy resulting from parental phubbing.

Conversely, frequent parental phubbing may disrupt the balance between the family system as a whole (i.e., family intimacy) and its subsystems (i.e., parent–child attachment). In such contexts, children may experience emotional neglect, as their needs for attention and responsiveness are insufficiently fulfilled, leading to reduced family intimacy. Diminished opportunities for meaningful parent–child communication may evoke feelings of rejection and insecurity, ultimately weakening attachment quality and impairing children’s social–emotional competence.

### 4.5 Limitations and future orientation

This study has several limitations that should be acknowledged. Firstly, other family contextual variables, such as socioeconomic status and parental educational attainment, were not controlled for. These factors may also influence children’s social–emotional competence, and future research could benefit from incorporating these variables as covariates.

Secondly, the cross-sectional design of this study does not permit causal inferences among the variables and cannot fully exclude reverse causality (e.g., young children’s low social–emotional competence eliciting parental phubbing). Longitudinal or experimental designs are recommended to further clarify the directionality of the observed relationships.

Thirdly, the data were collected through parental self-reports at the same time point, which may be subject to common method bias. Future research could incorporate additional measurement approaches, such as observational methods or third-party assessments, to obtain more objective evaluations of children’s social–emotional competence.

Finally, the survey sample was limited to children and parents from specific regions, with restricted geographical coverage. Future research could include samples from different regions and cultural backgrounds to conduct comparative analyses of the impact of cultural differences.

## 5 Conclusion

Parental phubbing influences children’s social–emotional competence through both the independent mediating roles of family intimacy and parent–child attachment and the serial mediating pathway linking these two factors. This serial mediation effect highlights the complex and interconnected processes within the family system through which parental behaviors shape children’s social–emotional development.

## Data Availability

The raw data supporting the conclusions of this article will be made available by the authors, without undue reservation.
